# Association between normal-weight obesity and cardiometabolic risk factors among adults in Addis Ababa, Ethiopia

**DOI:** 10.1038/s41598-023-49039-8

**Published:** 2023-12-20

**Authors:** Samson Gebremedhin, Mulugeta Mekonene, Seifu Hagos, Kaleab Baye, Bilal Shikur, Adugnaw Berhane, Tilahun Bekele

**Affiliations:** 1 School of Public Health, Addis Ababa University, Addis Ababa, Ethiopia; 2 Sport Science Academy, Wollo University, Dessie, Ethiopia; 3 Center for Food Science and Nutrition, Addis Ababa University, Addis Ababa, Ethiopia

**Keywords:** Diabetes, Cardiology, Diseases, Endocrinology, Medical research, Risk factors

## Abstract

The relationship between normal-weight obesity (NWO)—high percent body fat (%BF) in individuals with normal body mass index (BMI)—and cardiometabolic abnormalities has not been explored in Africa. We determined the prevalence of the NWO and evaluated its association with hypertension, elevated blood sugar and dyslipidaemia among adults in Addis Ababa, Ethiopia. A cross-sectional study was conducted among adults 18–64 years (n = 600). Blood pressure, blood glucose, lipid profile, and anthropometric measurements were completed. As a function of skinfold thickness, body density and %BF were estimated using Durnin & Womersley and Siri Equations, respectively. The relationship between the NWO and the outcomes of interest, assessed using adjusted linear and logit models. The age- and sex-standardised prevalence of NWO was 18.9% (95% confidence interval (CI) 15.8, 22.2%). Comparison between normal-weight lean (normal %BF and BMI) and normal-weight obese individuals suggested no difference in systolic blood pressure (β = 2.55; 95% CI − 0.82, 5.92); however, diastolic blood pressure (β = 3.77: 95% CI 1.37, 6.18) and odds of hypertension (adjusted odds ratio (AOR) = 2.46: 95% CI 1.18, 5.13) were significantly raised in the latter. Similarly, adults with NWO had elevated blood glucose (β = 2.30; 95% CI 1.23, 15.66) and increased odds of high blood sugar level (AOR = 1.68; 95% CI 1.05, 2.67). LDL (β = 8.73: 1.56, 15.90), triglyceride (β = 20.99: 0.78, 41.22), total cholesterol (β = 10.47: 1.44, 19.50), and Cholesterol to HDL ratio (β = 0.65: 0.27, 1.04) were also raised among adults with NWO. NWO is common among adults in Addis Ababa and is associated with cardiometabolic derangements.

## Introduction

Non-communicable diseases (NCDs) annually cause 41 million deaths, equivalent to more than two-third of all global deaths^1^. Low- and middle-income countries (LMIC) disproportionally take more than three-fourth of NCD-related deaths^1,2^. Four clinical conditions—cardiovascular diseases, cancers, chronic respiratory disease and diabetes mellitus—alone contribute to 80% of all NCD-related deaths^1^. Globally between 2000 and 2019, the total adult mortality attributable to NCDs has increased by 31%^3^.

Overweight including obesity, defined as accumulation of excessive or abnormal fat, is an established risk factor for hypertension, insulin resistance, dyslipidemia and several types of cancers^1,4,5^. Annually nearly 4 million people die as the consequence of high body mass index (BMI)^6^. In the last three decades the global prevalence of overweight including obesity has increased from 29 to 38% and obesity-related deaths and disability-adjusted life years (DALYs) have doubled^6–9^. In Ethiopia 3.1% of men and 7.6% of women are either overweight or obese^10^.

BMI is the most frequently used metric for classifying overweight and obesity. However, BMI does not differentiate the fat mass from lean mass, causing misclassification of individuals with high adiposity as normal^11,12^. Recently, normal-weight obesity (NWO)—a condition characterized by excess fat in individuals with normal BMI—is receiving increasing attention^12–14^. It has been reported that the “invisible obesity” is common and is linked with multiple cardiometabolic disfunctions^15–19^. Though there is no universally agreed threshold for excess percent body fat (%BF)^12,20^, studies from in India^15^ and China^16^ came across with high rates of NWO. Normal-weight obese, as compared to normal-weight lean individuals, have higher risks of metabolic syndrome and dying from cardiovascular conditions^17,18,21^.

Studies have suggested that the patterns of obesity and obesity-related comorbidities considerably vary across different populations^22–24^. However, to the best of our knowledge, the epidemiology and cardiometabolic consequences of NWO have not been investigated in an African population before. In this study, we determined the prevalence of NWO and explored its association with hypertension, elevated blood sugar and dyslipidaemia among adults in Addis Ababa, Ethiopia. Prior to the study, we hypothesized that increase in %BF even within normal BMI, increases the risk of metabolic syndrome.

## Methods

### Study design and setting

We used data from the baseline survey of the SuNCD-AA (Surveillance of Non-communicable Diseases in Addis Ababa) project designed to monitor the epidemiology of chronic diseases in the city on a 5-yearly basis using the WHO’s STEPwise Approach to NCD Risk Factor Surveillance (STEPS)^25^. The SuNCD-AA baseline survey was completed in June 2021.

Addis Ababa, is the capital and largest city of Ethiopia and has an estimated population of 4.5 million, of which 68% are adults 18–64 years of age^26^. Administratively Addis Ababa is divided into 10 sub-cities and 116 districts and has 12 public hospitals, 40 private hospitals, 96 health centres and more than 800 clinics. In 2006, a STEPS survey in the city reported high prevalence of overweight and obesity (29%), low physical activity (25%) and hypertension (30%) among adults 25–64 years of age^27^.

### Study population and design

In the SuNCD-AA survey, women and men 18–64 years of age, who were permanent residents of the city were eligible for inclusion irrespective of their medical history. Women participants were excluded if they had a self-reported pregnancy or gave birth in the preceding 12 months of the survey.

The baseline survey enrolled 600 adults 18–64 years of age. The original sample size was estimated using Cochran's single population proportion formula^28^ assuming 95% confidence level, 4% margin of error, 21% expected prevalence of overweight and obesity and design effect (DEFF) of 1.5. DEFF of 1.5 was determined using the standard DEFF = 1 + δ (n − 1) formula taking cluster size (n) of 20 and intra-cluster correlation of (δ) of 2%. Post-hoc analysis indicated, the available sample size was adequate to estimate 18.9% prevalence of NWO with 95% confidence level, 4% margin of error and DEFF of 1.5.

Study subjects were selected using multistage cluster sampling approach. Initially, from each of the 10 sub-cities 1 district, and subsequently from each district 2 villages (“*ketena*”), were drawn using the lottery method. Ultimately, 20 villages were represented in the study. In each of villages, using urban health extension workers’ database as a sampling frame, 20 households were selected at random. In each of selected household eligible subjects were listed, and one was selected using simple random sampling technique. Few selected individuals who were not willing to take part in the study or could not be found at home after repeated visits had been replaced with randomly selected eligible subjects from adjacent households.

### Variables of the study

The explanatory variable, NWO, was defined as having a normal BMI (18.5–24.9 kg/m^2^) with high %BF. As there is no universally agreed excess body fat threshold^12,20^, we primarily used the age- and sex-specific cut-offs (≤ 20th percentile of the norms) proposed by the American College of Sports Medicine (ACSM) for adults 20 years or older^29^. The ACSM thresholds that we used were: > 23.1 to 28.4% for men and > 27.1 to 35.4% for women, according to age^29^. As the ACSM provided no thresholds for adolescents 18 or 19 years of age, we applied the norms for young adults (20–29 years) to this group.

We have also provided alternative NWO prevalence figures based on the following three sex-specific %BF thresholds commonly used in the literature: (1) American Council on Exercise (ACE) threshold (≥ 25% for men and ≥ 32% for women)^30^, (2) ≥ 25% for males and ≥ 35% for females^31^ and (3) ≥ 20·6% for men and ≥ 33·4% for women^15,32^.

The primary outcomes of interest were blood pressure, blood glucose level and lipid profile (low-density lipoprotein (LDL), high-density lipoprotein (HDL), triglyceride and total cholesterol). The primary outcomes were analysed both as continuous and dichotomous variables. We considered age and sex as key control variables.

Based on JNC 7 classification^33^, hypertension was defined as a systolic blood pressure (SBP) of 140 mmHg or more, or a diastolic blood pressure (DBP) of 90 mmHg or above, or currently on medication for raised blood pressure. Base on the recommendation of the American Diabetic Association, we defined elevated blood sugar by aggregating prediabetic (Fasting Blood Sugar (FBS) between 100 and 125 mg/dl or Postprandial Blood Sugar (PPBS) between 140 and 199 mg/dl) and diabetic (FBS ≥ 126 mg/dl or PPBS ≥ 200 mg/dl or on medication for raised blood sugar) states^34^. Elevated LDL (≥ 100 mg/dl), triglyceride (≥ 150 mg/dl) and total cholesterol (≥ 200 mg/dl), and low HDL (< 40 mg/dl) were defined using clinically relevant cut-off values. Total cholesterol to HDL ratio was calculated as a composite index of dyslipidaemia. Seven individuals who reported that they were on anti-lipid treatment in the proceeding one week of the survey were excluded from lipid analysis.

### Data collection tools and procedures

Data were collected following the STEPwise Approach, a standardized method for monitoring behavioural, dietary and metabolic risk factors of NCDs^25^. The STEPS questionnaire was translated into Amharic language, pretested and contextualized to the local setting. Questions extracted from the standard Demographic and Health Survey (DHS) questionnaire were used to collect socio-demographic information. Physical activity level was measured using the Global Physical Activity Questionnaire (GPAQ) incorporated into the STEPS tool and classified as high, moderate or low based on the level of metabolic equivalent of task (MET)-minutes per week^25^.

Data were digitally collected using the Open Data Kit (ODK)^®^ system via KoBo Toolbox^®^ platform. Enumerators and supervisors were trained nurses with extensive field experience. The training included standardization of anthropometric measurements and procedures for measuring blood pressure and blood sugar levels.

### Anthropometric measurements

All participants underwent weight, height, waist and hip circumferences and skinfold measurements in the field following standard procedures. Weight was measured to the nearest 100 g without shoes and heavy clothing using SH2003B^®^ digital scale (accuracy ± 100 g). Standing height was measured without shoes to the nearest 0.1 cm using a portable Heuer^®^ stadiometer. BMI was calculated as weight in kilograms divided by height in meters squared and classified as underweight (< 18.5), normal (18.5–24.9), overweight (25.0–29.9) or obese (≥ 30).

Waist and hip circumferences were measured to the nearest 0.1 cm using a non-stretchable flexible tape with minimal clothing. Waist circumference was classified as normal (men < 94 cm and women < 80 cm), increased risk (men 94–102 cm and women 80–88 cm) or greatly increased risk (men > 102 cm and women > 88 cm)^35^. Waist-to-hip ratio (WHR) was classified as normal (men < 0.90 and women < 0.85) or substantially increased (men ≥ 0.90 and women ≥ 0.85)^35^.

All anthropometric measurements were performed in duplicate and, if the difference was within a tolerable range (200 g for weight, 0.5 cm for height, and 1 cm for waist and hip circumferences), the average was used. Otherwise, the measurements were repeated.

### Skinfold measurement and estimation of gross body composition

Dual-energy X-ray absorptiometry (DXA) is considered as the gold standard for measuring body fat. However, its applicability in community-based studies of low-income countries is limited to due to cost and feasibility reasons. In this study, we estimated body fat using the validated Durnin and Womersley Equation based on four skinfold measurements^36^.

Triceps, biceps, subscapular and suprailiac skinfold thickness measurements were completed in duplicate to the nearest millimetre using Plicometro^®^ callipers. Body density (g/ml) was predicted based on Durnin and Womersley Equation as a function of four skinfold measurements^36^. Though multiple equations have been proposed to estimate %BF using skinfold measurements, the Durnin and Womersley Equation has showed the strongest concordance with DXA measurements^37^. According to the equation, ${\text{Bodydensity}}\left( {\frac{{\text{g}}}{{{\text{ml}}}}} \right) = x - \left( {y\log 10L} \right)$ where: L is the total of the skinfolds (mm) and x and y are age- and sex-specific constants. Then %BF was determined using the Siri equation^38^, $\% {\text{BF}} = (495/D) - 450,$ where D is the density of the body (g/ml) estimated using Durnin and Womersley Equation. Ultimately, fat body mass (FBM) was computed by multiplying the total bodyweight by %BF and lean body mass (LBM) was determined by subtracting FBM from the total bodyweight.

### Lipid profile, blood pressure and blood sugar measurements

Blood pressure was measured using Folee^®^ automated digital monitor system in a sitting position after 15 min of rest. In individuals with recent exercise, smoking, heavy meal or caffeine intake, the measurement was delayed for at least 30 min. The measurement was repeated twice, and if the difference was within acceptable limit (10 mmHg for SBP and 5 mmHg for DBP) the average was recorded. Otherwise, a new set of readings was taken. Random blood glucose level was determined from capillary blood using the Diavue^®^ monitoring system. Lipid panel including, including low-density lipoprotein (LDL), high-density lipoprotein (HDL), triglyceride and total cholesterol were determined at the national reference laboratory of the Ethiopian Public Health Institute (EPHI).

### Statistical analysis

SPSS version 25 was used for data processing and analysis. Survey weights were applied to adjust for differences in probability of selection and to align the sample with known age and sex profile of the population. Survey weights were determined as a product of design weight and poststratification weight. Design weight was computed as the inverse of the sampling fraction. Poststratification weight was determined based on the age and sex composition of the city as reported in the recent national census^26^.

Categorical variables are expressed using frequency distributions. The normality of numeric variables was first assessed using Kolmogorov–Smirnov test and then appropriate measures of central tendency and dispersion were used to summarize the data. The arithmetic mean (± standard deviation (SD)) and median (inter-quartile range (IQR)) were applied to normal and skewed distributions, respectively. For proportions, 95% confidence interval (CI) was estimated using STATA’s binomial CI calculator. We also used a series of Pearson’s chi-square and chi-square tests for trend tests to compare proportions across two or more levels.

The association between the NWO and the outcomes of interest was measured by comparing normal-weight lean (normal %BF and BMI) and normal-weight obese individuals. Simple and multiple linear regression models were fitted for continuous outcomes (SBP, DBP and blood sugar level) and unstandardized regression coefficients (β) were used for interpretation. For dichotomous outcomes (hypertension, elevated blood glucose level, low HDL and elevated LDL, triglycerides and cholesterol) bivariable and multivariable logistic regression models were fitted and crude (COR) and adjusted (AOR) odds ratio were reported. All multivariable models were adjusted for age and sex.

### Ethical considerations

The study was conducted in accordance with the principles of the Declaration of Helsinki. The protocol was cleared by Institutional Review Board of College of Health Sciences, Addis Ababa University (109/20/SPH, 17/03/2021). Data were collected after taking informed written consents from the study participants.

## Results

### Basic characteristics

Data were obtained from 600 adults 18–64 years of age. With a male to female ratio of 0.93, women were slightly over represented. The mean (± SD) age of the participants was 31.2 (± 11.4) years and more than two-third were younger than 40 years. The vast majority (93%) had formal education, nearly half (46.8%) were married and more than two-thirds were employed at the time of the survey. The median (IQR) monthly household income was 4000 (2500–7000) Ethiopian Birr (equivalent to 95 (59–167) USD) and the median household size was 4 (3–5) (Table 1).

**Table 1:** Socio-demographic characteristics of the survey participants, Addis Ababa, Ethiopia, June 2021.

Socio-demographic characteristics (n = 600)	Frequency	Percentage
Sex		
Men	290	48.3
Women	310	51.7
Age (years)		
18–24	214	35.6
25–39	256	42.6
40–54	97	16.2
55–64	33	5.5
Educational status		
No formal schooling	42	7.0
Primary education	169	28.2
Secondary education	249	41.5
Higher education	140	23.3
Marital status		
Married/Cohabiting	281	46.8
Not ever married	269	44.8
Widowed	20	3.3
Divorced/separated	31	5.1
Occupation		
Not working (including retired)	182	30.4
Trade (including petty trade)	107	17.9
Student	102	17.0
Professional/technical/managerial	91	15.1
Manual (skilled or unskilled)	78	13.0
Others	39	6.6
Religion		
Orthodox Christian	486	81.1
Muslim	92	15.3
Protestants	19	3.1
Others	4	0.6

### Anthropometry and gross body composition

The mean (± SD) BMI was 24.1 (± 4.4) kg/m^2^ and 38.7% of the adults were either overweight or obese (BMI ≥ 25.0). The mean waist circumference was 81.8 (± 13.9) cm in men and 80.7 (± 13.9) cm in women; whereas, the mean waist-to-hip ratio was 0.89 (± 0.12) and 0.83 (± 0.12) in the two genders, respectively. Based on waist circumference, nearly one in three adults had increased or greatly increased risk and based on waist-to-hip ratio index, 40.9% had a substantially increased risk (Table 2).

**Table 2:** Anthropometric profile of adults 18–64 years in Addis Ababa, Ethiopia, June 2021.

Anthropometric profile	Men (n = 290)		Women (n = 310)		Both (n = 600)	
	Freq	%	Freq	%	Freq	%
Body mass index (kg/m^2^)						
Underweight (< 18.5)	34	11.6	8	2.6	42	7.0
Normal (18.5–24.9)	163	56.3	163	52.6	326	54.4
Overweight (25.0–29.9)	76	26.4	102	32.7	178	29.7
Obese (≥ 30)	17	5.7	37	12.1	54	9.0
Waist circumference						
Normal	236	81.6	157	50.5	393	65.5
Increased risk	35	12.2	72	23.2	107	17.9
Greatly increased risk	18	6.3	82	26.3	100	16.6
Waist to hip ratio (cm)						
Normal	175	60.6	179	57.7	354	59.1
Substantially increased	114	39.4	131	42.3	246	40.9

The mean predicted density of the body based on Durnin and Womersley Equation was 1038 (± 17) kg/m^3^ and the average %BF was 27.0%. The estimated prevalence of obesity based on %BF was 51.8% (30.5% in men and 71.8% in women). Fat mass (kg) and %BF were higher in women and fat free mass (kg) was higher in men (Table 3).

**Table 3:** Gross body composition among adults 18–64 years in Addis Ababa, Ethiopia, June 2021.

Gross body composition indices	Men (n = 290)	Women (n = 310)	Both (n = 600)
Mean (± SD) predicted body density (kg/m^3^)	1049 (± 13)	1027 (± 12)	1038 (± 17)
Mean (± SD) percent body fat (%)	21.8 (± 6.2)	31.9 (± 5.9)	27.0 (± 7.8)
Mean (± SD) fat mass (kg)	14.3 (± 6.3)	18.8 (± 6.5)	16.6 (± 6.8)
Mean (± SD) fat free mass (kg)	49.2 (± 7.7)	38.8 (± 5.9)	43.8 (± 8.6)
Body weight (kg)	63.5 (12.5)	57.5 (11.1)	60.4 (12.2)
Obesity estimated based on %BF	30.5	71.8	51.8

### Prevalence of normal-weight obesity

**Figure 1:**
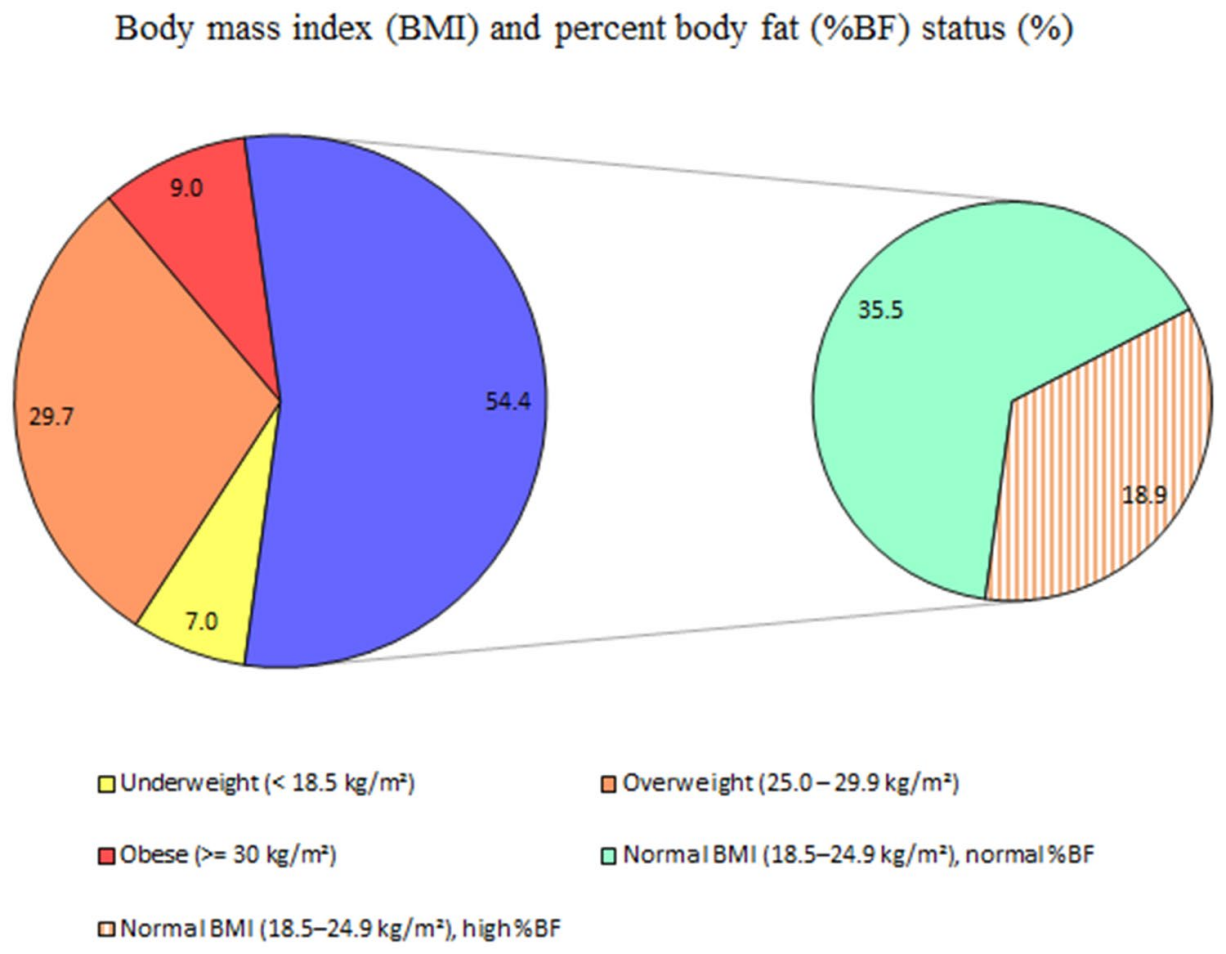
Prevalence of normal-weight obesity among adults 18–64 years, Addis Ababa, Ethiopia, June 2021.

Based on the %BF thresholds recommended by ACSM (> 23.1–28.4% in men and > 27.1–35.4 in women, according to age), the age- and sex-standardized prevalence of NWO among adults in Addis Ababa was 18.9% (95% CI 15.8–22.2%). Among adults with normal BMI, 34.8% (95% CI 30.0–40.1%) had NWO (Fig. 1).

**Figure 2:**
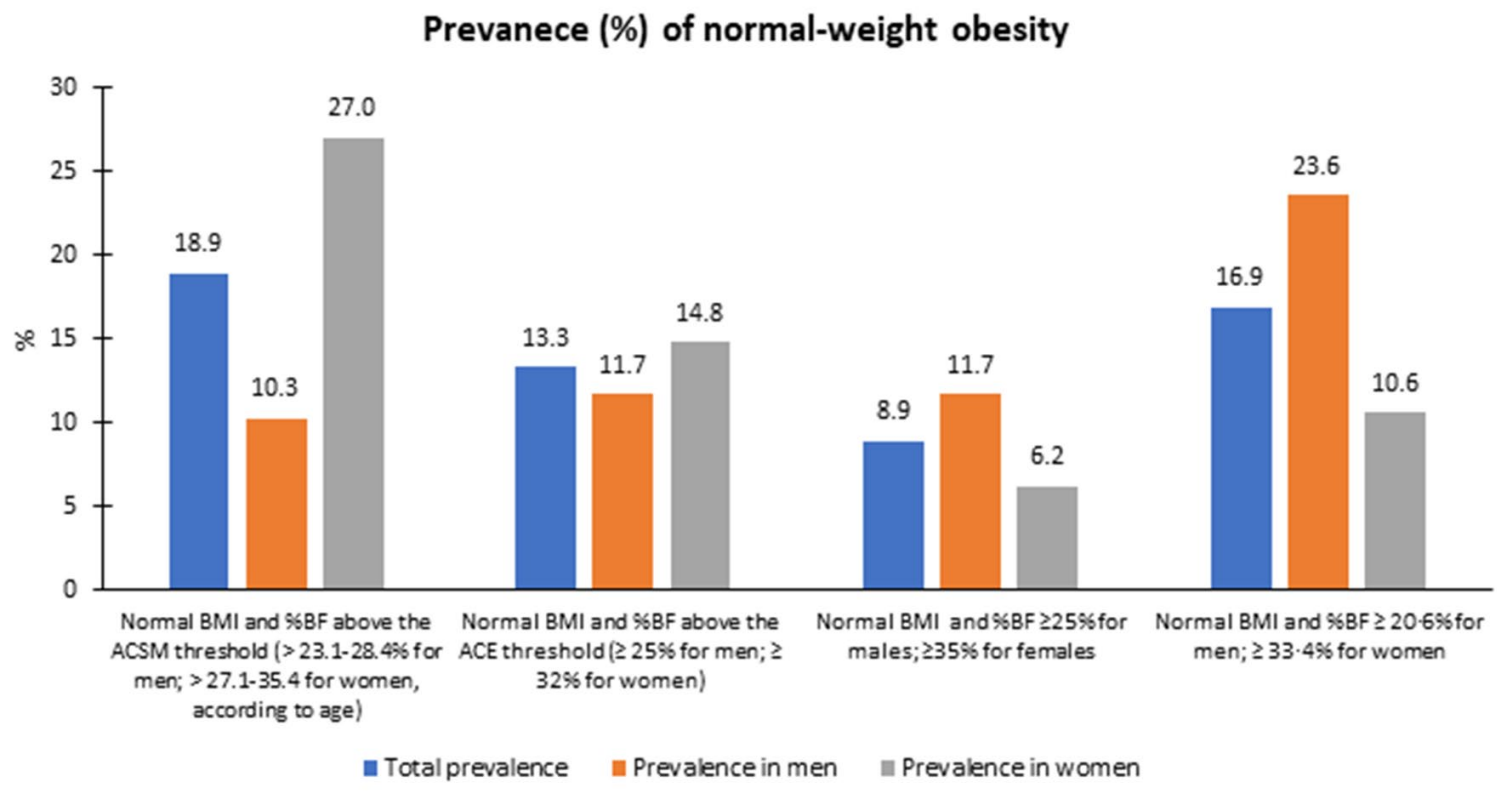
Prevalence of normal-weight obesity among adults in Addis Ababa as determined using four different thresholds for excess adiposity.

Figure 2 depicts the prevalence of the NWO as per four different %BF thresholds commonly used in the literature. The use of the ACSM age- and sex-specific limits provided the highest prevalence of 18.9%. Conversely, %BF threshold of ≥ 25% for men and ≥ 35% for women yielded the lowest prevalence of 8.8%. The choice %BF threshold differentially affects the sex-specific prevalence of NWO as well (Fig. 2).

We assessed the pattern or NWO (defined using the ACSM threshold) status across levels of sex, age and physical activity. NWO prevalence varied considerably between women (27.0%) and men (10.3%) (*p* = 0.0144), but did not significantly differ across age categories, 20.0% and 15.3%, among adults 18–39 and 40–64 years, respectively (*p* = 0.113). Regarding physical activity, the prevalence of NWO decreased from 28.4% in adults with low physical activity to 17.6% and 15.3% among those with moderate and high physical activities levels, respectively (*p* = 0.001).

### Association between normal-weight obesity and blood pressure, blood glucose and lipid profile

One-in-five (22.1%) had elevated blood pressure or were on hypertension treatment. Similarly, 23.2% had diabetes or prediabetes status and 16 (2.6%) were on treatment for elevated blood glucose in the previous 1 month of the survey.

Multiple linear regression analysis detected no significant difference in SBP (β = 2.55; 95% CI − 0.82, 5.92) between normal-weight lean and normal weight obese adults. However, DBP was significantly raised in the latter (β = 3.77; 95% CI 1.37, 6.18). Multivariable logit model adjusted for age and sex also indicated that adults with NWO had nearly twofold increased odds of hypertension (AOR = 2.46; 95% CI 1.18, 5.13).

Similarly, adults with NWO had significantly higher blood glucose concentration (β = 2.30; 95% CI 1.23, 15.66) and increased odds of elevated glucose level (AOR = 1.68, 95% CI 1.05–2.67) (Table 4).

**Table 4:** Association of normal-weight obesity with blood pressure, dyslipidaemia and blood sugar levels among adults 18–64 years, Addis Ababa, Ethiopia.

Continuous outcomes: mean (± SD)	Normal-weight obesity	Normal-weight lean	Linear regression^†^ (β with 95% confidence interval)	
			Crude	Adjusted^§^
Systolic blood pressure (mmHg)	116.8 (± 16.1)	115.8 (± 14.1)	1.05 (− 2.34, 4.44)	2.55 (− 0.82, 5.92)
Diastolic blood pressure (mmHg)	79.3 (± 11.4)	75.9 (± 9.8)	3.45 (1.08, 5.82)*	3.77 (1.37, 6.18)*
Blood sugar level (mg/dl)	115.1 (± 32.7)	106.4 (± 27.8)	8.62 (1.86, 15.38)*	7.28 (0.70, 13.87)*
Low-density lipoprotein (LDL) (mg/dl)	94.1 (± 31.2)	84.9 (± 28.1)	9.19 (2.49, 15.89)*	8.73 (1.56, 15.90)*
High-density lipoprotein (HDL) (mg/dl)	42.9 (± 13.0)	44.5 (± 11.3)	− 1.60 (− 4.32, 1.13)	− 2.76 (− 5.61, 0,10)
Triglyceride (mg/dl)	143.2 (± 102.5)	138.8 (± 78.3)	4.43 (− 15.60, 24.46)	20.99 (0.78, 41.22)*
Total cholesterol (mg/dl)	160.6 (± 41.6)	151.0 (± 34.7)	9.60 (1.08–18.13)*	10.47 (1.44–19.50)*
Cholesterol to HDL	4.08 (± 2.37)	3.57 (± 1.15)	0.51 (0.13, 0.90)*	0.65 (0.27, 1.04)*

Categorical outcomes (%)	Normal-weight obesity	Normal-weight lean	Logistic regression^†^ (odds ratio with 95% confidence interval)	
			Crude	Adjusted^§^
Hypertension (%)	19.5	11.3	1.95 (1.04, 3.67)*	2.46 (1.18, 5.13)*
Elevated blood sugar level (%)^‡^	23.9	12.7	2.18 (1.21–3.95)*	2.03 (1.11–3.73)*
Elevated LDL (≥ 100 mg/dl) (%)	37.2	19.2	2.48 (1.49, 4.14)*	2.52 (1.42, 4.47)*
Low HDL (< 40 mg/dl) (%)	61.9	64.3	1.13 (0.70, 1.81)	1.24 (0.73, 2.11)*
Elevated triglyceride (≥ 150 mg/dl) (%)	32.7	30.5	1.12 (0.69–1.83)	2.08 (1.14–3.78)*
Elevated cholesterol (≥ 200 mg/dl) (%)	12.4	7.0	1.87 (0.87–4.04)	2.72 (1.17–6.32)*

*Statistically significant association at P value of 0.05.

^†^Codded as:0 = normal weight obesity absent, 1 = normal weight obesity present.

^§^Adjusted for age and sex.

^‡^ Includes prediabetic and diabetic blood sugar levels.

We also compared the lipid profile between adults with and without NWO. Multiple linear regression model adjusted for age and sex detected no statistically significant difference in HDL concentration (β = − 2.76: − 5.61, 0.10). However, LDL (β = 8.73: 1.56, 15.90), triglyceride (β = 20.99: 0.78, 41.22) and total cholesterol (β = 10.47: 1.44–19.50) were all significantly raised among adults with NWO. The odds of elevated LDL (AOR = 2.52: 1.42, 4.47), triglyceride (AOR = 2.08: 1.14–3.78) and cholesterol (AOR = 2.72: 1.17–6.32) were all raised among adults with NWO than their counterparts.

Cholesterol to HDL ratio among adults with NWO (4.08 ± 2.37) was also significantly lower in adults with normal BMI but raised %BF (3.57 ± 1.15), (β = 0.65: 0.27, 1.04) (Table 4).

## Discussion

We examined the prevalence of the NWO and its association with cardiometabolic risk factors. We found NWO is fairly common in adults from Addis Ababa and more frequently occurs among women and adults with low physical activity levels. Our study suggested significant association between NWO and hypertension, dyslipidaemia and elevated sugar level.

After applying age- and sex-specific %BF thresholds (> 23.1–28.4% in men and > 27.1–35.4 in women, according to age), we found that nearly one-in-five adults in Addis Ababa had NWO. In South Korea^32^ and India^15^, based on sex-specific thresholds (≥ 20·6% for men, ≥ 33·4% for women), 32% prevalence of NWO was reported. In Colombia, using sex-specific cut-offs (> 25.5% for men, > 38.9% for women) 29% of adults 18–30 years had NWO^39^. It is important to note that the prevalence figures reported across the studies cannot be directly compared due to differences in %BF thresholds applied to define excess adiposity. Yet, the studies suggested that NWO is common in many countries including LMIC.

The high prevalence of NWO that we observed among adults in Addis Ababa suggested that in clinical and research contexts, assessment of cardiometabolic risk factors solely based on BMI may lead to considerable misinterpretation of adiposity. Routine assessment of body fat enables researchers and practitioners to differentiate normal-weight adults with and without excess adiposity.

We observed considerable differences in the crude and sex-specific prevalence of NWO as defined based on four adiposity thresholds. In Switzerland, the prevalence of NWO varied noticeably according to the cut-off used to define %BF^20^. Reviews conducted on the topic have also documented the same^12,31^. The finding implies that there is an obvious need for developing optimized excess adiposity thresholds for different populations.

According to the results, a sizable difference in the prevalence of NWO was observed between men (10.3%) and women (27.0%). Though women are known to have more body fat than men, the natural difference should have been offset by the use of sex-specific thresholds. In this regard, the available studies have so far reported conflicting findings^17,32,39^. In Colombia, the prevalence in women (46%) was substantially higher than in men (2.0%)^39^; but the opposite was true among Korean adults (36 and 29% in men and women, respectively)^32^. On the other hand, the magnitude was fairly balanced between Brazilian men (9.2%) and women (9.0%)^17^. The higher prevalence of NWO that we observed in women can be either due to elevated true risk in women (e.g., low physical activity) or inadequate optimization of the %BF thresholds to counterbalance the natural adiposity difference between the two genders.

We observed an inverse association between NWO and levels of physical activity. The finding is consistent with the understanding that greater physical activity results in lower adiposity irrespective of BMI^40,41^. A secondary analysis of the UK Biobank Assessment Centres’ data concluded that physical activity was inversely associated with adiposity even among people with the same BMI^40^. In China, similar pattern of association was also observed among older adults (40–64 years) with BMI below 24 kg/m^2^^41^. The finding implies that, on top of the proven benefit for optimizing BMI, physical activity can reduce adiposity among adults with normal BMI.

Our study showed NWO was associated with significantly increased odds of hypertension (OR = 2.5) and raised blood sugar level (OR = 1.7). Reports from US^18,42^, Asian^15,16,19^ and Latin American^17,39^ populations have indicated that an increase in adiposity within normal BMI range raises the risk of insulin resistance and/or hypertension. According to a study from India, NWO increased risk of diabetes but not hypertension^15^. In the US, the association with hypertension was only limited to men^18^.

We found that NWO was associated higher cholesterol to HDL ratio and increased odds of elevated LDL (AOR = 2.5), triglyceride (AOR = 2.1) and cholesterol (AOR = 2.7). However, it was not associated with HDL level. In Korean^32^ and Indian^15^ adults, NWO has been also associated with atherogenic lipid profile. A cohort study in Brazil found that low HDL and high triglyceride were common among Brazilian young adults^17^. A study among Colombian young adults (18–30 years) reported that the prevalence of high LDL was 29% and 21% in NWO and NWL adults respectively, but the difference did not reach statistically significant levels^39^. Low HDL level was more common among adults with NWO^39^.

The uniqueness of this study is that it assessed the epidemiology of NWO in African setting where no study has been done on the topic so far; furthermore, it assessed the association of NWO with multiple outcomes including elevated blood sugar, hypertension and dyslipidaemia. However, the study has to be interpreted with consideration of the following limitations. (1) The ACSM thresholds applied to define excess adiposity had not been validated to the setting and this may have caused an over- or under-estimation of NWO. (2) We estimated %BF using a validated equation as a function of multiple skinfold measurements; however, this method is not accurate as DXA or bioelectrical impedance methods. (3) With the intension of maximizing our sample size, individuals who have not consented to study or could not be found at home were replaced with others from the neighbourhood. This may have introduced selection bias in the study. (4) Ideally blood glucose and lipid profile have to be measured in fasting state; however, in the current study, random blood samples were used and this may cause misclassification bias. (5) RBS was determined with glucose meter using capillary blood; however, plasma glucose determination using venous blood would have been more appropriate. (6) While assessing the association between NWO and blood sugar level, we aggregated prediabetes and diabetes states together because the number of diabetic cases were too small for meaningful analysis. This may have introduced a misclassification bias in the study.

## Conclusion

Normal-weight obesity is common in adults from Addis Ababa and more frequently affects women and adults with low physical activity level. In the population that we studied, NWO is associated with increased risk of hypertension, hyperlipidaemia and elevated sugar level. The study implies, screening of cardiometabolic risk factors solely based on BMI may lead to considerable underestimation of adiposity and its consequences.

## Data Availability

The datasets used during the current study are available from the corresponding author on reasonable request.

## Abbreviations

%BF: Percent body fat
AOR: Adjusted odds ratio
ACE: American Council on Exercise
ACSM: American College of Sports Medicine
BMI: Body mass index
COR: Crude odds ratio
CI: Confidence interval
DALYs: Disability-adjusted life years
DBP: Diastolic blood pressure
DEFF: Design effect
DHS: Demographic and Health Survey
DXA: Dual-energy X-ray absorptiometry
EPHI: Ethiopian Public Health Institute
FBS: Fasting blood sugar
GPAQ: Global Physical Activity Questionnaire
IQR: Inter-quartile range
HDL: High-density lipoprotein
LDL: Low-density lipoprotein
LMIC: Low- and middle-income countries
MET: Metabolic equivalent of task
NCDs: Non-communicable diseases
NWO: Normal-weight obesity
ODK: Open Data Kit
PPBS: Postprandial Blood Sugar
SBP: Systolic blood pressure
SD: Standard deviation
STEPS: STEPwise Approach to NCD Risk Factor Surveillance
SuNCD-AA: Surveillance of Non-communicable Diseases in Addis Ababa
WHR: Waist-to-hip ratio
